# Using Chemical Reaction Kinetics to Predict Optimal Antibiotic Treatment Strategies

**DOI:** 10.1371/journal.pcbi.1005321

**Published:** 2017-01-06

**Authors:** Pia Abel zur Wiesch, Fabrizio Clarelli, Ted Cohen

**Affiliations:** 1 Department of Epidemiology of Microbial Diseases, Yale School of Public Health, New Haven, Connecticut, United States of America; 2 Centre for Molecular Medicine Norway, Nordic EMBL Partnership, Oslo, Norway; 3 Department of Pharmacy, Faculty of Health Sciences, University of Tromsø, Tromsø, Norway; University of New South Wales, AUSTRALIA

## Abstract

Identifying optimal dosing of antibiotics has proven challenging—some antibiotics are most effective when they are administered periodically at high doses, while others work best when minimizing concentration fluctuations. Mechanistic explanations for why antibiotics differ in their optimal dosing are lacking, limiting our ability to predict optimal therapy and leading to long and costly experiments. We use mathematical models that describe both bacterial growth and intracellular antibiotic-target binding to investigate the effects of fluctuating antibiotic concentrations on individual bacterial cells and bacterial populations. We show that physicochemical parameters, e.g. the rate of drug transmembrane diffusion and the antibiotic-target complex half-life are sufficient to explain which treatment strategy is most effective. If the drug-target complex dissociates rapidly, the antibiotic must be kept constantly at a concentration that prevents bacterial replication. If antibiotics cross bacterial cell envelopes slowly to reach their target, there is a delay in the onset of action that may be reduced by increasing initial antibiotic concentration. Finally, slow drug-target dissociation and slow diffusion out of cells act to prolong antibiotic effects, thereby allowing for less frequent dosing. Our model can be used as a tool in the rational design of treatment for bacterial infections. It is easily adaptable to other biological systems, e.g. HIV, malaria and cancer, where the effects of physiological fluctuations of drug concentration are also poorly understood.

## Author Summary

In this era of rising concerns about antibiotic resistance, the rational design of optimal antibiotic treatment regimens remains an important unrealized goal. At this time, the characteristics of antibiotic treatment regimens (e.g. dosing levels, treatment duration, route of administration) are determined largely based on costly in vivo experiments. The sheer number of possible dosing strategies that must be tested contributes to the delay and cost of the development of new drugs and may limit the feasibility of finding optimal regimen characteristics. Here, we demonstrate how modeling the chemical kinetics of drug-target binding can identify the best time-concentration profile of antibiotics. Using both analytical approaches and numerical simulations, we find that the physicochemical characteristics of drug-target binding are sufficient to explain the pharmacodynamics of commonly used antibiotics such as ampicillin, isoniazid and tetracycline. In practical terms, our models can be used as a tool in the rational design of treatment for bacterial infections. Because of the generality of drug-target binding kinetics, these approaches may also be adapted to other diseases where the effects of physiological fluctuations of drug concentration are also poorly understood, such as HIV, malaria and cancer.

## Introduction

The rise of antibiotic resistance underlines the need for employing existing antibiotics prudently. Although antibiotic dosing regimens have been investigated for more than half a century [1], we do not yet have a sufficient understanding of the link between drug dosing and bacterial killing to design rational treatment strategies [2, 3]. Even for antibiotic regimens that have been standard of care, substantial improvements in dosing levels [4], treatment frequency [5] and treatment duration[6–8] have been made decades after their introduction. Most experimental and some clinical studies investigate antibiotic concentrations at a constant or at an average concentration. However, drug concentration at target tissues can fluctuate substantially over time. These fluctuations can influence the effectiveness of treatment, with the importance of such fluctuations differing substantially between classes of antibiotics [9].

**Fig 1:**
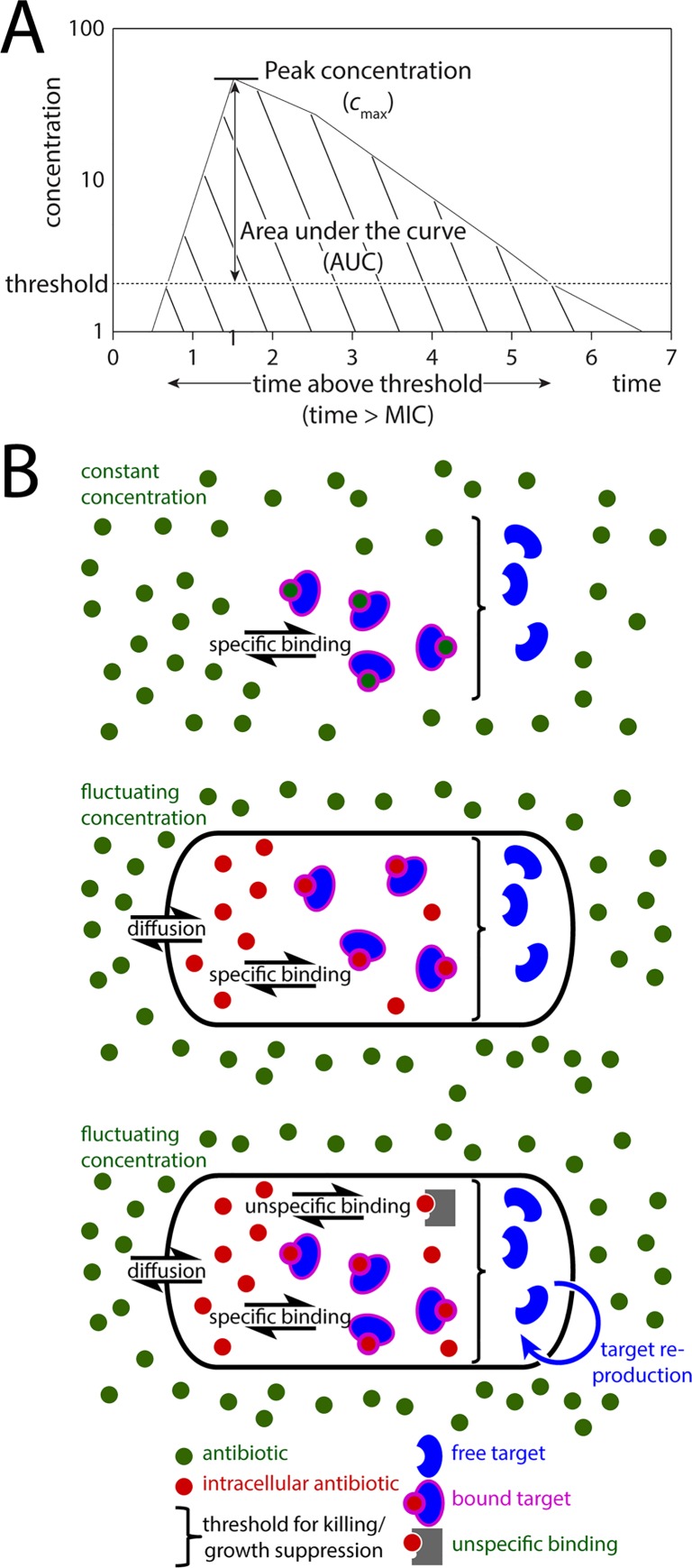
Schematic overview of descriptors of effective drug concentration and the used models. **(A).** This graph shows the *in vivo* concentration profile after drug ingestion in arbitrary units. Three descriptors are commonly used to predict efficacy: The time above a specific threshold, commonly the MIC (dashed line, *T*_C>MIC_), the peak concentration (*C*_max_), or the area under the curve (AUC, hatched area). **(B)** Overview of the used models. Model 1 (upper panel) only follows the extracellular antibiotic concentration, bound and unbound target molecules and assumes that the antibiotic concentration remains constant over time during the period of administration. Model 2 (middle panel) allows for fluctuating antibiotic concentrations and a diffusion barrier between antibiotic molecules outside the cell and their intracellular targets. Model 3 (lower panel) incorporates the reproduction of target molecules through bacterial replication and considers unspecific binding of the antibiotic.

Three alternative descriptions of effective antibiotic concentration are commonly used (so-called pharmacokinetic drivers): i) the total concentration integrated over a given time interval (area under the curve, AUC), ii) the peak concentration (*C*_max_) or iii) the time during which the concentration exceeds a specific threshold (time above MIC, *T*_C>MIC_, Fig 1A). For some drugs *C*_max_ correlates best with bacterial clearance [10], for example in clinical trials with isoniazid [11]. Even once-weekly dosing was slightly superior to daily dosing for the novel TB drug bedaquiline when holding total drug administration constant [12]. For rifampicin [13] and quinolones, the total amount of drug [14] appears to be the best predictor of treatment success. For beta-lactams, the time above the minimal inhibitory concentration (MIC) correlates best with bacteriological response [2]. For some antibiotics, such as tetracycline, antibacterial action depends on both *T*_C>MIC_ and AUC [10]. Each of these three measures of exposure (AUC, *C*_max_ and *T*_C>MIC_) would be optimized by employing different dosing strategies, for example by using large intermittent doses to increase *C*_max_ or by employing extended release formulations to increase *T*_C>MIC_ [15].

A clear mechanistic understanding of antibiotic pharmacodynamics has not yet been achieved, and this lack of knowledge is a major obstacle for the design of rational treatment regimens. Treatment strategies for bacterial infections (e.g. dose levels, dosing frequency, and duration of therapy) are usually developed based on pharmacodynamic and pharmacokinetic data collected through expensive in vitro and in vivo studies [9, 16–18]. Specifically, the question of which pharmacokinetic driver governs antibiotic efficacy has to be determined experimentally with hollow-fiber systems or animal models [11, 19, 20]. This experimental information in turn can be incorporated into mathematical models [21], but to our knowledge there is no mathematical model that can guide these experiments.

Thus, the development of models that can inform optimal dosing strategies from data collected in early phases of antibiotic development could speed the drug development process and help to identify promising compounds that should be prioritized [22]. Here, we extend a modeling framework [23] that integrates bacterial population biology with the intracellular reaction kinetics of antibiotic-target binding to investigate how the kinetics of drug-target binding affect bacterial response to fluctuating antibiotic concentrations. We find that the physicochemical characteristics of drug action predict differences in antibiotic pharmacodynamics at fluctuating concentrations and correlate well with observed data.

## Results

Using three models that incorporate complexity and realism in a stepwise fashion (Fig 1), we consider how reaction kinetics govern the expected bacterial responses to antibiotics. First, we use a simple model that considers only drug-target binding to explore the general principles of antibiotic-target reaction kinetics. Then, we use more complex models to simulate the action of two specific antibiotics, ampicillin and tetracycline, under a range of different dosing strategies. We assess which physicochemical characteristics of these two drugs explain their distinct pharmacodynamic behavior and evaluate how an understanding of these physicochemical characteristics can inform more effective dosing regimens.

## Model 1: General Principles of Antibiotic-Target Reaction Kinetics

In this section, we will employ Model 1, which allows us to focus exclusively on the kinetics of antibiotic-target binding *A* + *T* ⇌ *AT* (Model 1, Eqs (1–5)). Using this framework, we first explore the relationship of the MIC with physicochemical parameters. We then investigate the factors that may cause a delay in the bacterial response to antibiotics after initial exposure and factors that may extend these responses after antibiotics are withdrawn.

### MIC defined by physicochemical parameters

**Fig 2:**
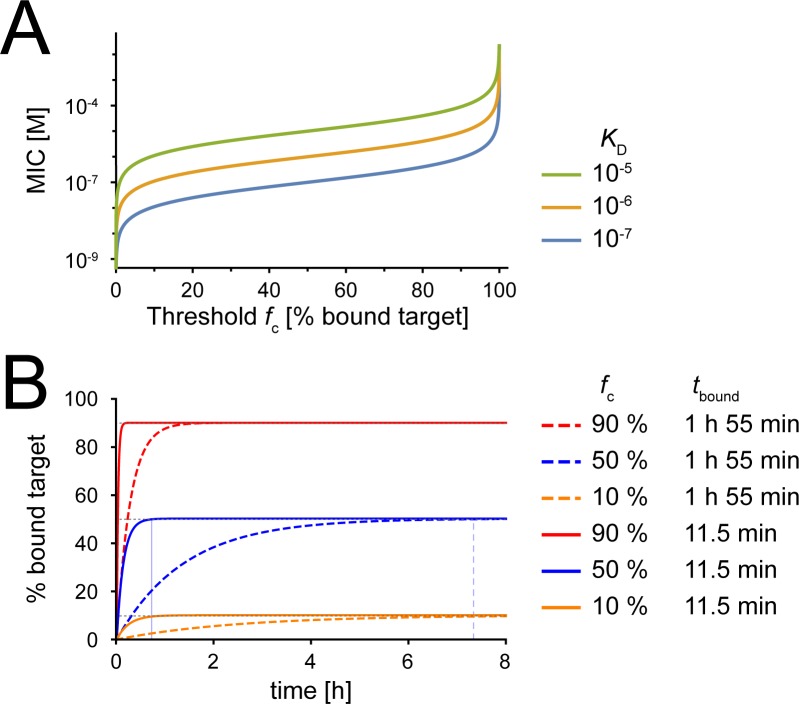
Delay to onset of antibiotic action depends on turnover rate and target occupancy at MIC. **(A)** A definition of the MIC based on physicochemical characteristics. This graph shows the expected MIC in mol/L (based on Eq (3), y-axis) as a function of target occupancy at MIC (*f*_c_, x-axis). The colors indicate different affinities of drug target binding (*K*_D_). Blue: *K*_D_ = 10^−7^ M, yellow: *K*_D_ = 10^−6^ M, green: *K*_D_ = 10^−5^ M. **(B)** This graph illustrates the time course of drug-target reaction (based on Eq (4)) for various parameter sets and a fixed antibiotic concentration just above MIC (1.01 x MIC). Dotted lines: Slow turnover rate of antibiotic-target binding with half-life of drug-target complex *t*_bound_ = 1h 55 min (*k*_r_ = 10^−4^). Solid lines: Fast turnover rate of antibiotic-target binding with half-life of drug-target complex *t*_bound_ = 11.5 min (*k*_r_ = 10^−3^). The colors indicate different target occupancies at MIC. Red: *f*_c_ = 90%, Dark blue: *f*_c_ = 50%, Yellow: *f*_c_ = 10%. The grey lines at 90%, 50% and 10% indicate the *f*_c_, the threshold of bound target required to kill/inactivate the cell. The light blue solid and dotted vertical lines indicate when the fast and slow reactions reach the *f*_c_, i.e. the time of onset of the antibiotic action (*t*_onset_).

Recommended antibiotic dosage varies widely depending on the employed antibiotic and the targeted pathogen. It is therefore difficult to compare antibiotic action in terms of absolute concentrations. Typically, all measures of antibiotic efficacy are defined relative to the MIC of the specific bacteria/drug pair (*C*_max_/MIC, AUC/MIC and *T*_C>MIC_) to circumvent this problem. To be able to use a modeling framework based on physicochemical characteristics of drug action, it is therefore useful to define the MIC based on physicochemical properties [23]. In the simplest case, when we assume a constant antibiotic concentration in this framework, the MIC depends on two parameters: the drug target affinity (*K*_D_) and the threshold of bound target (*f*_c_) at which the net growth of a bacterial population is zero (Eq (3)). Fig 2A illustrates the expected MIC according to Eq (3) which depends on drug target affinity *K*_D_ and the critical threshold *f*_c_. The absolute concentration of antibiotic at MIC rises with the threshold occupied target required for bacterial suppression (*f*_c_). Given any threshold level of target occupancy, drugs with a higher binding affinity (lower *K*_D_) will require smaller concentrations to prevent bacterial growth (lower MIC).

### Onset of antibiotic action

**Fig 3:**
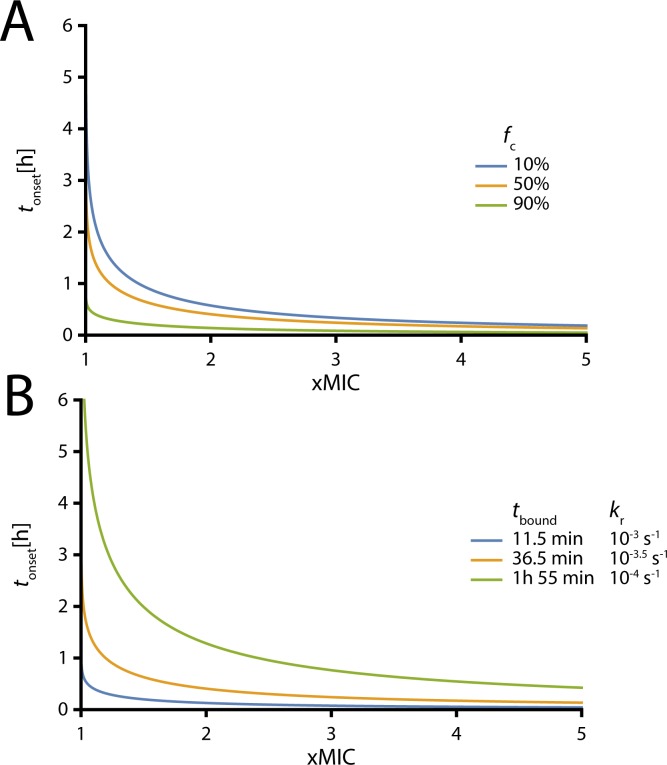
The benefit of high drug concentrations depends on the velocity with which the reaction reaches equilibrium. These graphs show the dependence of the onset of antibiotic action as measured in time required for reaching the threshold *f*_c_ on the drug concentration in fold-MIC. It is based on Eq (5). **(A)** Illustration of scenarios with different thresholds *f*_c_ with a constant drug target half-life *t*_bound_ of 36.5 min (*k*_r_ = 10^−3.5^). Blue: *f*_c_ = 10%, yellow: *f*_c_ = 50%, green: *f*_c_ = 90%. **(B)** Illustration of scenarios with different drug target half-lives *t*_bound_ with a constant threshold *f*_c_ = 50%. Blue: *t*_bound_ = 11.5 min (*k*_r_ = 10^−3^), yellow: *t*_bound_ = 36.5 min (*k*_r_ = 10^−3.5^), green: *t*_bound_ = 1h 55min (*k*_r_ = 10^−4^).

Classical models of antibiotic pharmacodynamics typically assume that the antibiotic concentration at any time point determines the net bacterial growth rate at that same time point. This assumes both that the antibiotic acts instantaneously and that previous antibiotic exposure has no continuing influence on bacterial growth. In reality, however, there is typically a delay between initial exposure and antibiotic effect and there may also be post-antibiotic period in which bacterial growth remains suppressed even after the antibiotic is removed from the extracellular space. Here we use our modeling framework to understand how the onset and end of antibiotic action are affected by the physicochemical properties of drug-target binding. We use the reaction kinetics of drug target binding (Eq (4)) to show the dynamics when the antibiotic is applied at a concentration slightly above MIC (1.01 x MIC). We use this concentration for illustration purposes because at this concentration, the critical fraction of binding *f*_c_ is reached in finite time, but never substantially exceeds this threshold. (The influence of higher concentrations is explored in Fig 3.)

The different scenarios in Fig 2B illustrate the time course of drug-target binding at the same concentration relative to the MIC, but different absolute antibiotic concentrations. From the limited number of studies in which antibiotic-target dissociation rates have been directly measured, we assume that these rates range between 10^−3^/s and 10^−4^/s [24, 25]. Slower turnover of drug-target binding (i.e. a longer half-life of the drug-target complex) is associated with a delayed onset of action (compare Fig 2B dotted to solid lines).

Surprisingly, we find that the system approaches equilibrium more quickly when *f*_c_ is higher. This effect can be explained as follows: The absolute antibiotic concentration at MIC rises sharply with the threshold *fc*, and can map to very different absolute drug concentrations (see Fig 2A). Initially, only the forward reaction is relevant when a negligible amount of target is bound and proceeds with the rate *k*_*f*_[*A*][*T*_0_] (i.e. as the product of forward reaction rate, antibiotic concentration and target molecule concentration). Under conditions where the antibiotic concentration is held constant, the equilibrium fraction of bound target [A] is given by ${\left[AT\right]}_{eq}=\frac{\left[A\right]}{[A]+K_{D}}$ and asymptotically approaches 1. Therefore, the velocity of the reaction increases more quickly than the fraction of bound target at equilibrium. This produces a paradoxical finding: when dosing antibiotics at the same levels relative to their respective MIC, those that require a high threshold of bound target to be effective are expected to act more quickly (Fig 2B).

The delay until an antibiotic is effective depends on many physiological and biochemical factors. Since this model focuses on the reaction kinetics alone (ignoring diffusion barriers and concentration gradients), Model 1 provides a lower bound for the expected delay until onset of antibiotic action. Even here, for reasonable parameter settings, we find that even this delay can extend for several hours. One potential approach for speeding antibiotic-target binding and reducing delay to onset of action is to increase antibiotic exposure through higher dosing. Lower thresholds and slower turnover are associated with delays until antibiotic action; these effects can be overcome by increasing the drug concentration (Fig 3). The light blue solid and dotted vertical lines in Fig 2B indicate when the fast and slow reactions reach the *f*_c_, i.e. the time of onset of the antibiotic action (*t*_onset_), and can be compared to the blue and green lines in Fig 3B at 1.01MIC. When the antibiotic-target reaction equilibrates slowly, a high dose of antibiotic is especially beneficial and minimizes the opportunity for additional bacterial replication events prior to onset of antibiotic action.

### End of antibiotic action

Bacterial growth often remains suppressed after the antibiotic concentration drops below the MIC (i.e. the post-antibiotic effect). This effect occurs because drug-target complex dissociation is not instantaneous. Therefore, high drug concentrations that saturate the target beyond the threshold required for antibiotic action *f*_c_ may have additional benefits if they extend bacterial suppression beyond the time that the antibiotic concentration exceeds the MIC.

**Fig 4:**
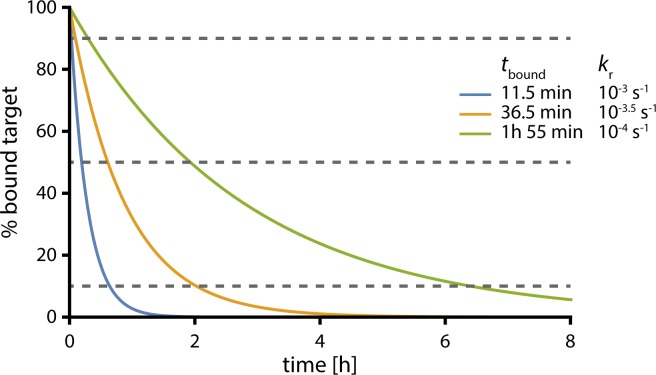
Turnover rate and target occupancy at MIC determine end of antibiotic action. This graph illustrates the time course of drug-target dissociation after 99.9% of the target was bound according to an exponential decay $\frac{\left[AT\right]}{\left[AT]+[T\right]}=99.9\;e^{-k_{r}t}$. Blue: *t*_bound_ = 11.5 min (*k*_r_ = 10^−3^), yellow: *t*_bound_ = 36.5 min (*k*_r_ = 10^−3.5^), green: *t*_bound_ = 1h 55min (*k*_r_ = 10^−4^). The grey lines at 90%, 50% and 10% illustrate when the fraction of bound target falls below a particular threshold *f*_c_.

We use our model to identify the conditions in which high antibiotic concentrations are expected to prolong antibiotic action. For simplicity, for these simulations we assume that at the time of antibiotic withdrawal, 99.9% of the target is bound and that the antibiotic concentration both inside and outside of the bacterial cell immediately drops to zero. Under these assumptions, Eq (1) can be simplified and the unbinding of the antibiotic corresponds to a simple exponential decay. Fig 4 illustrates the expected dissociation of the drug-target complex for antibiotics with different half-lives *t*_bound_. When the threshold required for antibiotic action *f*_c_ is very high, the antibiotic stops working very rapidly and the length of the post-antibiotic effect is brief and relatively insensitive to the half-life of the drug-target complex. Conversely, when there is both a low threshold and a slow turnover time of drug-target binding, the post-antibiotic period may last for several hours.

## Model 2: Onset and End of Antibiotic Action Determine Pharmacodynamic Properties of Ampicillin and Isoniazid

Next, we investigate the dynamics of drug target binding under different dosing regimens. We use ampicillin as an example, because a large body of literature describes the time-dependent action of beta-lactam antibiotics, both in experimental models as well as in patients [26–30]. In addition, the reaction kinetics of drug-target binding are relatively well established. To investigate the generality of our findings, we then simulate the reaction kinetics of isoniazid, a prodrug that accumulates in the bacterial cell.

### Model 2a: Ampicillin

Beta-lactams acetylate penicillin-binding proteins (PBPs, the target molecules), and thereby inhibit cell wall synthesis. The acetylation of PBPs consumes beta-lactams, and therefore the drug-target reaction is not reversible. However, PBPs are constantly de-acetylated and the effects of the antibiotic are therefore reversible. The kinetics of PBP acetylation and de-acetylation as well as target occupancy at MIC have been determined experimentally (Table 1). In single cell experiments, ampicillin has no detectable sub-MIC activity (S1 Fig) so we assume that antibiotic is effective only while the fraction of bound antibiotic exceeds *f*_c_.

**Table 1:** Parameters and references.

Target	Antibiotic	Parameter	Value	References
Ribosome	-	Copy #/cell	55000 [~10^4^−10^5^]	[31–33]
	Tetracycline	*K*_D_^a^^)^	1.1–3.2 x 10^−6^ M	[34]
		*k*_f_ ^b^^)^	3 x 10^5^ M^-1^ s^-1^	[35]
		*k*_r_ = *K*_D_ x *k*_f_ ^c^^)^	0.33–0.96 s^-1^	
		*f*_c_	0.98	[23]
		*p*	1.2 x 10^−2^ s^-1^	[23]
Penicillin binding proteins (PBPs)	-	Copy #/cell	~ 2500 ± 120	[36, 37]
	Ampicillin^d^^)^	Irreversible		
		*k*_f_	130 ± 1 M^-1^ s^-1^	[25]
		Deacetylation rate (*k*_a_)	1 x 10^−4^ s^-1^	[25]
		*f*_c_	0.954	[38]
		*p*		Location outside cytosol- assumption: no diffusion barrier
enoyl acyl carrier protein reductase (inhA) ^e^	Isoniazid (all values for M. tuberculosis)	rate of drug activation (INH +NAD -> INH-NAD)^8)^	1.8 x 10^−6^ s^-1^	[39]
		K_D_	10^−7^ M	[40]
		k_f_ = k_r_/ K_D_	2.8 x 10^3^ M^-1^ s^-1^	[24][24][24][24]
		k_r_	2.8 x 10^−4^ s^-1^	[41]
		**f**_**c**_	0.6	[23]
		h (length M.tb)	3 um	[42]
		R (width M.tb)	0.35 um	[42]
		L (thickness cell wall M.tb)	15 nm	[43]
^f^		**D**_**w**_ (diffusion coefficient through M.tb cell wall)	1·10^−10^ dm^2^s^-1^	[44, 45]

^a^Tetracycline binds reversibly to six sites in the ribosome. However, one primary target site binds most strongly and is most responsible for inhibition of translation [34, 46, 47]. For 70S particles, the equilibrium constant K_D_ for this site is in the range of 1.1–3.2 μM, depending on Mg^2+^ concentration.

^b^The apparent association rate of tetracycline (summing over several binding sites) to the ribosome was estimated to be ~3 x 10^5^ M^-1^ s^-1^ [35]. For simplicity, we assume here that the primary binding site alone is responsible for antimicrobial activity and its association rate is equal the apparent association rate.

^c^The dissociation rate was to our knowledge never directly measured, and we calculated it from the *K*_D_ and the association rate.

^d^Binding rates for PBP1a are given. In *Staphylococcus aureus*, the binding rates did not differ substantially between different classes of PBPs, so we assume that the binding rates are equal for all PBPs [38].

^e^For both InhA and the drug-activating enzyme, KatG, the number of molecules per cell is unknown. Instead, we chose the average number of proteins in *E*. *coli* cells from [48].

^f^ The flux throughout the cell wall is $J=-D_{w}\frac{\partial [C]}{\partial x}\approx -\frac{D_{w}}{L}({\left[C\right]}_{e}-{\left[C\right]}_{i})$; and the permeability p is: $p=\frac{D_{w}}{L}\;\frac{A}{V_{i}}\;\left(\frac{1}{sec}\right)$ where *A* is the cell surface and *V*_*i*_ the intracellular volume.

**Fig 5:**
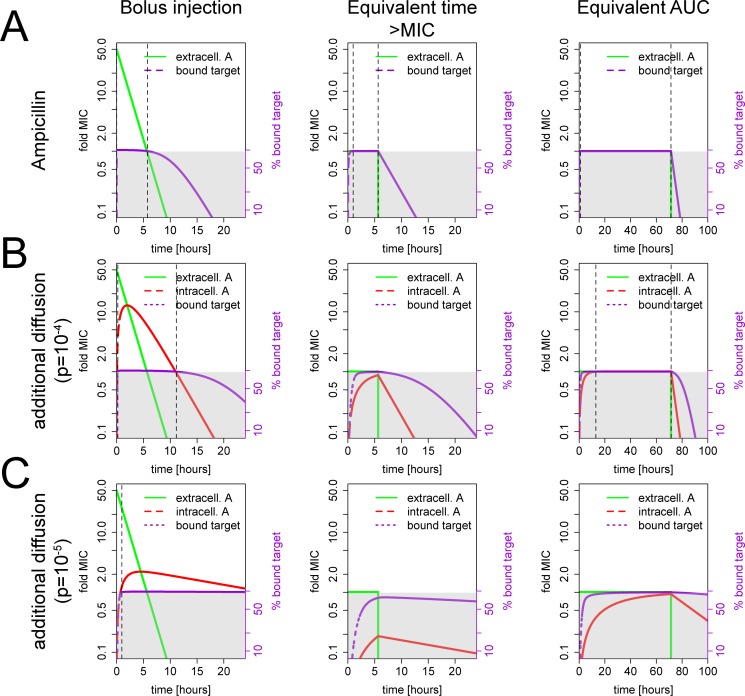
Biochemical properties shape antibiotic pharmacodynamics. These graphs show the expected dynamics of antibiotic-target reaction according to model 2 with all parameters adapted to ampicillin except stated. The x-axes show the time after initiation of antibiotic therapy in hours, the y-axes the current antibiotic concentration in fold MIC (black, left side) and the % bound target (violet, right side). Note that the y-axis is on a logarithmic scale. The green line shows the antibiotic concentration outside and the red inside the cell (both refer to the y-axis on the left), the violet line shows the amount of bound target (refers to y-axis on the right). The grey area indicates that either the antibiotic concentration is below MIC or the fraction of bound target is below the inhibitory threshold *f*_c_. The dotted vertical lines indicate beginning and end of antibiotic action. Graphs in the first column depict bolus injections with an initial antibiotic concentration of 50MIC and a half-life of 1h. The second column shows a hypothetical dosing regimen with a constant concentration just above the MIC (1.01 MIC) that has the same *T*_C>MIC_ as in the first column. The third column shows a hypothetical dosing regimen with a constant concentration just above the MIC (1.01 MIC) that has the equivalent area under the curve (AUC) as in the first column. Note the different timescale in the third column. **(A)** Biochemical properties are sufficient to explain time-dependent action of beta-lactams. The graphs show drug-target binding expected based on physicochemical characteristics of ampicillin drug-target binding from the literature (Table 1). **(B)** Area under the curve is best predictor of antibiotic action for equilibration times in the range of hours. We introduced a diffusion barrier of *p* = 10^-4^s^-1^ while all other parameters remain as in (A). **(C)** Peak concentration is best predictor of drug action when equilibration is slow. We introduced a stronger diffusion barrier of *p* = 10^-5^s^-1^ while all other parameters remain as in (A).

To explore whether *T*_C>MIC_, AUC or *C*_max_ are the best predictors of antibiotic efficacy, we model three simplified dosing strategies: i) an idealized bolus injection where the drug concentration immediately reaches its peak and then declines exponentially, ii) a hypothetical pharmacokinetic curve where the antibiotic concentration is maintained just above the MIC (1.01x MIC) for the same length of time >MIC as in i) and then falls instantaneously to 0, and iii) a curve of similar shape to ii) that retains the same area under the curve as i) (see Fig 5A). In ii), the time above MIC is identical to i) but we eliminate the excess binding that occurs because of the initial high peak in i). In iii), the AUC is the same as in i) but instead of the high peak concentration, there is a significantly prolonged *T*_C>MIC_. In other words, all graphs in the middle column of Fig 5 have the same time > MIC as those in the left column, and all graphs in the right column have the same AUC as those in the left column.

First, we investigate whether our modeling framework can reproduce the time-dependent action of beta-lactams based on the known physicochemical characteristics of the drug and its target. Fig 5A shows numerical simulations of Eq (6) using experimentally determined parameters (see Table 1). Note that this equation includes diffusion across the bacterial cell envelope. However, penicillin-binding proteins are either located in the cell envelope or in case of gram-negatives in the periplasm, and we therefore assume here that the diffusion barrier to the target is negligible. We adapted Eq (6) to describe the consumption of beta-lactams during target acetylation by dropping the backward reaction term *k*_*r*_[*AT*] for the differential equation describing the intercellular antibiotic [*A*]_i_. For all three dosing strategies, antibiotic action starts immediately after the drug concentration rises above MIC and stops as soon as the drug concentration falls below MIC, i.e. increasing the AUC alone without increasing *T*_C>MIC_ does not change antibiotic action substantially (compare Fig 5A left and right panel). This is in accordance with the observation that the efficacy of beta-lactams strongly depends on the time above MIC. Taken together, we can reproduce time-dependent action of beta-lactams solely based on reaction kinetics. The high threshold required for activity as well as the extracellular location of the target lead to a fast onset of drug action as the antibiotic concentration rises above the MIC and a nearly immediate end of antibiotic action as the antibiotic concentration drops below the MIC. To illustrate the different dynamics of bolus injections and constant dosing, we visualize time course of ampicillin action for a bolus injection in S1 Movie (Fig 5A, left panel) and for a constant concentration in S2 Movie (Fig 5A, middle panel).

Beta-lactams are the antibiotic class for which time-dependent action is most widely accepted, and we can reproduce their time-dependent action with our model. This suggests that physicochemical characteristics may be responsible for this behavior. For most other antibiotic classes, antibacterial efficacy is better correlated with AUC or *C*_max_ [13, 14]. We hypothesized that alteration in specific physicochemical parameters could generate AUC and *C*_max_-dependent action. To investigate this hypothesis, we modified the parameters for ampicillin one at a time to determine whether, through such parameter modification, we could reproduce AUC and *C*_max_-dependent action. Because antibiotic treatments are usually given over several days and the time between individual doses is typically in the range of hours, we first investigated parameter changes that produce an equilibration time of several hours. For example, if the drug must diffuse across a cell envelope with a diffusion rate of *p* = 10^−4^ /s, this leads to a half-life of free intracellular drug of 1h 55min. Fig 5B shows a comparison of the same dosing strategies as used for the upper panel (Fig 5A) with this additional diffusion barrier. With such a strong diffusion barrier the antibiotic concentration inside the cell also remains above MIC after a bolus injection of 50x MIC for several hours because antibiotic molecules are retained within the cell. Consequently, the activity after such a bolus administration can be extended by several hours (Fig 5B). However, the diffusion barrier also delays the onset of antibiotic action. This delay is dependent on the antibiotic concentration, the left panel of Fig 5B shows a delay of 14 minutes while the right panel shows a delay of 13h. This is because the equilibration of intra- and extracellular concentration is slower when there are smaller differences between the concentrations outside and inside the bacterial cell. If the antibiotic dose is only slightly above MIC, >10h are required to reach the threshold for inhibition *f*_c_ (Fig 5B, right panel). Thus, a dosing strategy with an equivalent time >MIC as the 50x MIC bolus administration will never achieve bacterial suppression, while a dose with an equivalent area under the curve is approximately 5.4 times more effective than the bolus injection (bolus injection: antibacterial activity from 14min to 11h9min, constant concentration with same MIC: activity from 13h to 71.5h). S2 Fig shows the dynamics when keeping *C*_max_ constant but varying the drug half-life. Again, we would expect beta-lactam action to start immediately and end immediately when the drug falls below MIC; however, for an antibiotic with a substantial diffusion barrier, we would expect delays until the onset and cessation of drug action.

This behavior is not limited to slow equilibration rates due to diffusion barriers. Earlier, we identified two parameters that affect the onset and the end of antibiotic action: target occupancy at MIC (*f*_c_) and the half-life of the drug-target complex (*t*_bound_). A long exposure to ampicillin at MIC is expected to result in a target saturation that just reaches the critical threshold *f*_c_ = 95.4% [38] at equilibrium. After withdrawal of the drug, the target saturation would immediately fall below this critical threshold and the antibiotic would no longer be active. The duration of antibiotic action might be extended with higher drug concentrations since this would produce higher target saturation and lead to a longer delay until the fraction bound target falls below the critical threshold *f*_c_. However, increasing the target saturation at the beginning from 95.4% to 99.9% is expected to extend the action of ampicillin only by about 9 minutes ($0.999\frac{ln(f_{c})}{-k_{r}}$). On the other hand, if the critical threshold *f*_c_ was 10% instead of 95.4%, achieving a target saturation of 99.9% would extend the expected time of antibiotic action by over 6h. Similarly, if the rate of de-acetylation is decreased 10-fold (*k*_r_ = 10^−5^), the expected duration of antibiotic action after achieving a target saturation of 99.9% is extended by over 1.5h. Changing both parameters to values that result in equilibration rates in the range of hours leads to the same qualitative behavior as when the equilibration rate is in the range of hours because of a diffusion barrier (S3 Fig). Thus, our model predicts that changing a single physiochemical parameter (equilibration times to the range of hours) has major impact on pharmacodynamics: instead of *T*_C>MIC_ alone, the AUC becomes another predictor of antibiotic efficacy and both are needed to predict antibiotic action. S1 Movie and S2 Movie illustrate the time course of the action of a hypothetical antibiotic that has the same binding and de-acetylation rates as ampicillin, but where the antibiotic must cross a diffusion barrier with *p* = 10^−4^ /s and a threshold of *f*_c_ = 10%. As in S1 Movie and S2 Movie, we compare a bolus injection (S3 Movie) and a concentration with the same time above MIC (S4 Movie).

We now examine the consequences of slow drug equilibration rates (i.e. in the range of days) on predicted antibiotic pharmacodynamics. We slow the diffusion rate across the bacterial cell envelope to *p* = 10^−5^, which corresponds to a half-life of 19h 15min. In this case, the antibiotic concentration inside the cell remains above MIC after a bolus injection of 50x MIC for a day. Exposure to the antibiotic at a concentration only slightly above MIC (1.01 x MIC) is insufficient to achieve the required amount of bound target, even when maintained for several days (Fig 5C). Thus, in situations where antibiotics are expected to equilibrate slowly, a high peak concentration is necessary to achieve antibiotic action and the *C*_max_ is expected to be the best predictor of antibiotic action.

### Model 2b: Isoniazid

We further tested this finding by investigating the reaction kinetics of a drug with a very different mechanism of action: the antitubercular pro-drug isoniazid (INH). In this case, target binding occurs after drug activation to the adduct INH-NAD which depends on NAD content and oxygen saturation. Importantly, the majority of the active drug INH-NAD remains in the mycobacterial cell and is not able to cross the cell envelope [49, 50].

**Fig 6:**
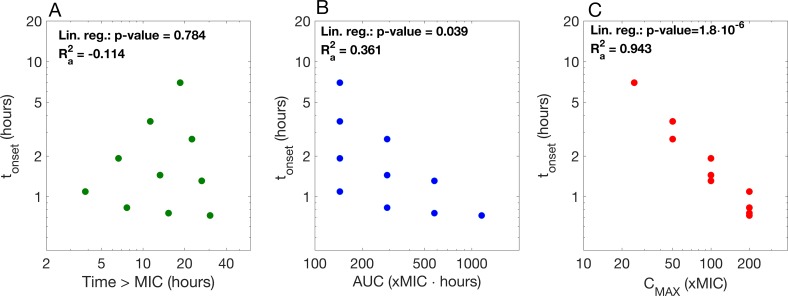
Correlation between pharmacokinetic drivers and time until average cells are predicted to killed with isoniazid. This graph shows the time until the threshold *f*_*c*_ is reached after simulated bolus injections (model 2b) of 5-100x MIC isoniazid (typical *C*_max_ values during therapy are at around 25x MIC [51, 52]) and a half-life of 0.5 – 4h. We used experimentally determined values for the MIC (0.1mg/l) for *M*. *tuberculosis* [53]. The different panels show the correlation between the time until the threshold is reached and different pharmacokinetic measures: (A) time above MIC; (B) area under the curve; (C) *C*_max_. We correlated the logarithm of *t*_*onset*_ with the logarithm of the three pharmacokinetic indices in single linear regressions.

Because INH-NAD remains in the cell, the expected amount of bound target does not decline even when the external concentration of INH declines. We therefore interpret treatment success here as the required time to reach *f*_*c*_, i.e. the expected time after which an average bacterium is killed. Again, we can reproduce experimental and clinical findings that INH treatment efficacy is significantly correlated with both *C*_*max*_ and *AUC* in univariate regressions (Fig 6). S1 Table gives an overview of all parameters combinations used in the simulations.

In a multivariate regression, only *C*_max_ is significantly correlated with the time to reach the required threshold to kill bacteria (S4 Fig), although the best model according to the Akaike Information Criterion includes all three pharmacokinetic indices. Due to the prodrug-activation, it takes several hours to reach the threshold required for killing, and this delay can be reduced with high peak concentrations. Since the active drug, INH-NAD, is trapped inside the cell, the intracellular drug concentration does not decrease when the external concentration decreases. Therefore, it is not necessary to keep the external pro-drug concentration above MIC for the drug to be active.

## Model 3: Antibiotic Action below MIC Modifies Pharmacodynamic Properties of Tetracycline

The time above the MIC is expected to be a reasonable predictor of antibiotic action in situations where antibiotic concentrations below the MIC have little effect on bacteria. For example, cells exposed to 80% MIC ampicillin show no measurable defect in either growth or elongation rates, and all cells remain intact (S1 Fig). In contrast, translation inhibitors such as chloramphenicol and tetracycline do affect bacterial growth below MIC, and it has previously been shown a nearly complete suppression of growth at 80% MIC [23]. When fitting Eq (10) to data from single cells exposed to constant sub-MIC concentrations of antibiotics [23], it has previously been estimated that a high threshold of bound ribosomes must be met to interrupt all bacterial growth (*f*_c_ = 98%) and that there is a low diffusion barrier (*p* = 1.2x 10^−2^/s). Experimental values from the literature suggest a short half-life of the drug-target complex (Table 1). Based on the values of these parameters, we expect that the *T*_C>MIC_ should be the best predictor of tetracycline effects. However, experimental and clinical evidence suggests that both AUC and *T*_C>MIC_ determine the efficacy for tetracycline [23]. Accordingly, we used Model 3 (Eq (10) populated with parameters for tetracycline), to investigate how sub-MIC activity affects antibiotic pharmacodynamics under different dosing strategies.

**Fig 7:**
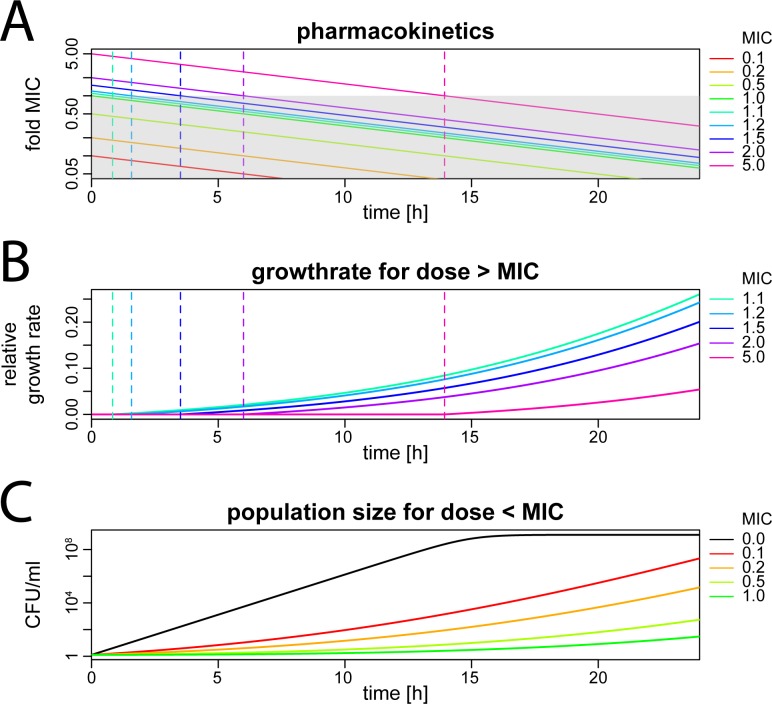
Antibiotic action of tetracycline used in different concentrations. These graphs show results of numerical simulations of Eq (10) parameterized with previously fitted values [23]. The x-axis shows the time in hours after antibiotic administration. The different colors indicate the initial antibiotic concentration in fold MIC (see legend). **(A)** Simplified pharmacokinetics (first-order clearance) of a tetracycline bolus injection with a half-life of 6h. The y-axis indicates the antibiotic concentration in fold MIC. The grey shaded area indicates an antibiotic concentration below MIC. The vertical dotted lines indicate when the antibiotic concentration for different dose levels falls below MIC. **(B)** Effect of supra-MIC doses on bacterial growth rate (y-axis). Again, the vertical dotted lines indicate when the antibiotic concentration for different dose levels falls below MIC. **(C)** Effect of sub-MIC concentrations of tetracycline on bacterial population size (y-axis).

Fig 7A shows simplified pharmacokinetics of a tetracycline bolus injection with initial concentrations ranging from 0.1–5 x MIC. Fig 7B shows the effects of dosages above MIC on the bacterial growth rate. Given the low diffusion barrier, bacterial growth is completely suppressed as long as the antibiotic concentration is retained above MIC. As soon as the antibiotic concentration falls below MIC, bacterial growth immediately resumes and continues to increase in rate as the antibiotic is cleared. Thus, when considering pharmacokinetic measures that correlate with the complete suppression of bacterial growth, the time above MIC is the best predictor of antibiotic action. However, the model also suggests that sub-MIC concentrations may substantially affect the total expected bacterial load over 24h (Fig 7C).

**Fig 8:**
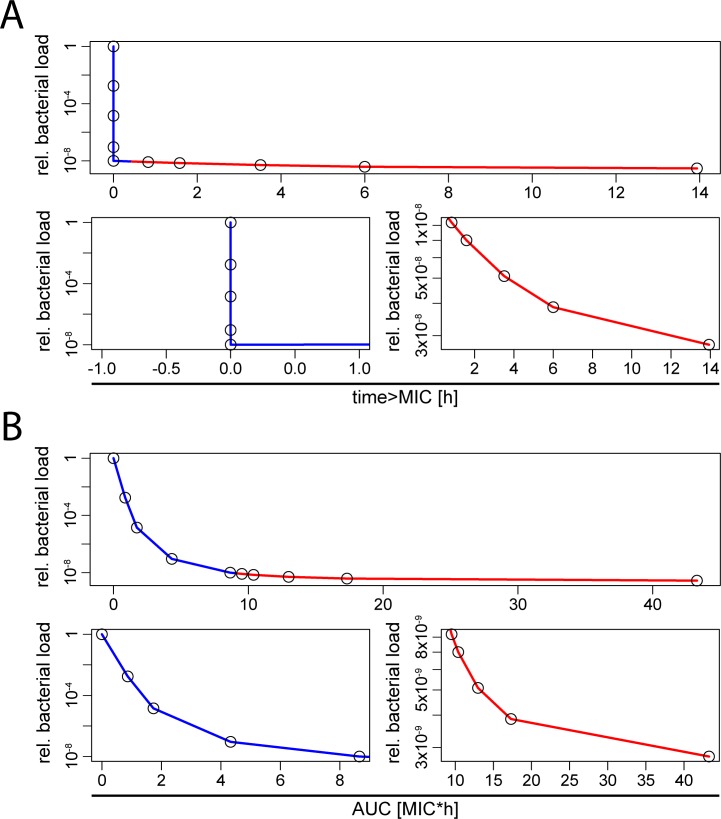
Time above MIC is insufficient predictor when sub-MIC concentrations are biologically active. These graphs show results from the numerical simulations shown in Fig 7. Antibiotic efficacy is measured in fold reduction of total bacterial load during 24h normalized to a bacterial population growing in the absence of antibiotics. Blue = peak concentration < MIC, red = peak concentration > MIC. The lower panels in (A) and (B) show sub- and supra-MIC concentrations separately for clarity. **(A)** This graph shows the correlation between *T*_C>MIC_ and antibiotic efficacy. **(B)** This graph shows the correlation between area under the curve and antibiotic efficacy.

Accordingly, our model predicts that *T*_C>MIC_ may be an imperfect predictor of antibiotic action (at least as measured by its effect on the total bacterial burden over 24h) since sub-MIC exposure can impact expected bacterial burden (first 5 data points in Fig 8A, highlighted in blue). In contrast, for antibiotic dosages above MIC, *T*_C>MIC_ correlates well with efficacy (last 5 data points in Fig 8A, highlighted in red), however, it should be noted that the overall effect is very small. Nevertheless, in a treated patient with low remaining bacterial burden the additional killing of few bacteria can make the difference between cure (i.e. extinction of bacterial population) or relapse. Our model is deterministic and therefore cannot capture extinction, however, depending on the initial population size a very small frequency of survivors that translates to less than one bacterium effectively means extinction. Over a wider range of antibiotic dosages in bolus injection, the area under the curve correlates more strongly with antibiotic effects because it is a measure that also reflects actions that occur below MIC (Fig 8B).

## Discussion

It is well known that certain pharmacokinetic measures (i.e. AUC, *C*_max_ or *T*_C>MIC_) are better predictors of the pharmacodynamics of some antibiotics than of others, but we currently have limited quantitative understanding of the mechanisms that drive this phenomenon. In this paper, we extend a model that links chemical reaction kinetics to bacterial population biology [23] and suggest a potential mechanistic explanation for this phenomenon.

Based on this model, we suggest how physicochemical and biochemical characteristics of drug-target interaction may shape antibiotic dose response curves. Differences in characteristics between antibiotics offer a compelling explanation for the observation that different measures of drug exposure correlate best with antibacterial activity. Specifically, we identified four factors that govern patterns of drug effects: i) the half-life of the antibiotic-target complex, ii) the diffusion barrier between extracellular antibiotic and its target, iii) the threshold of bound target required to suppress bacterial growth (i.e. target molecule occupancy at MIC) and iv) drug effects when the antibiotic is present only at sub-MIC levels.

The first three factors, the half-life of drug-target complex, the diffusion barrier and the threshold required for bacterial suppression, all influence the time until the antibiotic starts and stops acting (i.e. the equilibration rate of the reaction). When the onset of action of an antibiotic is rapid, we expect that achieving drug concentrations just above MIC should be sufficient to trigger the antibacterial effect. If an antibiotic stops acting quickly, antibiotic effects should cease as soon as the concentration falls below MIC. In these circumstances, we expect that the time above MIC would be a good measure for antibiotic efficacy. We demonstrated that our model, when parameterized with relevant drug-target binding data from the literature, can reproduce such time-dependent pharmacodynamics of ampicillin. Beta-lactams are somewhat unique in that their targets are located outside the cytosol [54]. Therefore, there is negligible diffusion barrier between the antibiotic molecules surrounding a bacterial cell and their targets. Our model predicts that this leads to a fast onset and end of antibiotic action. Also, almost all target molecules are occupied at MIC [38], and we demonstrate here that this also should lead to a rapid onset and cessation of antibiotic activity. Time-dependent efficacy of beta-lactams is well established both experimentally and clinically. For example, it is recommended that beta-lactams are given as continuous infusion rather than bolus injections [27].

For most other antibiotic classes, antibacterial efficacy is correlated with AUC or *C*_max_ [10, 14]. Many antibiotics have targets that are located in the cytosol (e,g. ribosomal-targeting antibiotics such as streptomycin or gyrase-targeting antibiotics such as ciprofloxacin). Also, unlike beta-lactams, many antibiotics will have effects before the majority of target molecules are bound. We therefore investigated whether our model can also reproduce concentration-dependent patterns of antibiotic action, in which antibiotic efficacy is best described by either *C*_max_ or *AUC*.

Indeed, our model predicts that the *T*_C>MIC_ is not highly correlated with treatment efficacy when the time until an antibiotic starts and stops being active (i.e. the equilibration time) is in the range of hours or longer. The delay until an antibiotic is effective depends on many physiological and biochemical factors. Here, we focus on the reaction kinetics alone, which provide a lower bound for the expected time to onset of antibiotic action. We note that even these lower-bound estimates may be as long as a few hours, potentially permitting several additional generations of bacterial replication. We would therefore suggest high doses, at least initially, for antibiotics that: 1) act at low thresholds of bound target; 2) diffuse only slowly through the cell envelope; or 3) have a slow turnover rate (i.e. a long half-life of drug-target binding). A similar argument can be made for the anti-tuberculosis drug isoniazid, which is a prodrug that is activated by bacterial cells. The activation rate of the drug alone is sufficient to explain the slow onset of action of the drug [23], and this delay can likely be reduced with higher antibiotic doses. Indeed, the efficacy of isoniazid has been linked to high peak doses [55], a finding we were able to reproduce here. Additionally, when equilibration rates are slow, higher dose of antibiotics can extend the action of the antibiotic beyond the time the antibiotic concentration outside the bacteria exceeds the MIC. Thus, high doses have the additional benefit of prolonging the post-antibiotic period for antibiotic-target pairs that equilibrate slowly. In isoniazid, this extension of drug action predicted by our model is especially pronounced, because the drug is trapped in the cell such that declining external drug concentrations have little effect. In principle, these delays in onset and end of action are a similar phenomenon to the concept of a “biophase lag” [56] although the underlying mechanisms are not the same.

To examine the conditions in which each of these pharmacokinetic metrics provides the best measure of drug effect, we compared a dosing strategy with a high peak concentration that facilitates rapid onset of an antibacterial effect with a dosing strategy that has an equivalent AUC, but a lower peak concentration and a substantially longer exposure time (Fig 5). If an antibiotic equilibrates slowly, the onset of antibiotic action at low doses is so delayed that the required fraction of bound target cannot be reached before the antibiotic falls below MIC in the low dose/long exposure strategy (Fig 5). Obviously, the exact parameter ranges in which this is the case depend on the definition of “long” (in our case, days). If equilibration is too slow compared to the relevant timeframe (for example due to the accumulation of activated isoniazid in the cell), we would expect that *C*_max_ is a better predictor of antibacterial efficacy than the AUC. Whether the peak concentration (*C*_max_) or the total exposure (*AUC*) is the best predictor of antibiotic efficacy thus depends on both the observed timeframe and the equilibration rate.

In addition to the onset and end of antibiotic action, we found that the biological activity of the antibiotic at sub-MIC concentrations also determines which pharmacokinetic measure best predicts treatment efficacy. A similar argument has been made for rifampicin therapy in tuberculosis[57]. Some antibiotics such as ampicillin (S1 Fig) have very little effect below MIC. In contrast, some antibiotics like tetracycline have some sub-MIC activity. Clearly, the time above MIC alone cannot predict treatment success when sub-MIC concentrations partially suppress bacterial growth. Indeed, our model predicts that treatment efficacy with tetracycline depends both on *T*_C>MIC_ and AUC which is in concordance with clinical and experimental studies [10].

Taken together, our mechanistic model can reproduce the pharmacodynamic characteristics of both ampicillin and tetracycline. It offers an intuitive explanation for differences in optimal dosing strategies between antibiotic classes. However, the parameters needed to inform even such a simple model have not yet been measured for many antibiotic/bacterial pairs. We note that most of the kinetic measurements for antibiotic-target binding were published decades ago [24, 34, 35]. To our knowledge, beta-lactams are the only antibiotic class for which target occupancy at MIC has been experimentally determined. Furthermore, the number of target molecules per cell and especially the concentration of free antibiotic at the target site are rarely known, despite being a focus of active research in tuberculosis [58–61]. We suggest that experiments to address these knowledge gaps should be prioritized as the results of these studies could inform new approaches for the rational dosing of antibiotics.

Identifying optimal antibiotic dosing strategies is challenging and in this paper we have addressed only a subset of the considerations that must be accounted for when determining treatment recommendations. For example, antibacterial efficacy and toxicity must be balanced and the frequency of dosing may affect adherence; these are important factors that should doubtless affect treatment recommendations. In addition, our simple models do not consider host immune responses to infection, which may further modify our expectations regarding treatment success [57, 62, 63]. Nevertheless, given the urgent need to preserve the efficacy of existing antibiotics and the need to develop new agents [64], we see a promising role for mechanistic models that can suggest the most promising dosing strategies based on the physicochemical and biochemical characteristics of drug-target interactions. Such novel pharmacodynamics models can also be integrated into more complex frameworks that include host responses and more sophisticated pharmacokinetics [57, 62, 63].

Our model is general and we believe it could be usefully adapted to improve dosing strategies for treatment of other diseases. For example, we note that the effects of the physiological fluctuations of drug concentration are also poorly understood in the treatment of cancer [65], HIV [66] and malaria [67] and similar questions arise regarding the effects of exposure to harmful substances in toxicology [68].

## Methods

Previously, we have shown that models that consider drug-target binding kinetics can explain complex patterns of antibiotic action such as post-antibiotic effects, inoculum effects, and persistence [23]. The central assumption of these models is that bacterial replication decreases and/or bacterial killing increases with the fraction of bound target molecules. Here, we extend this approach using three different mathematical models that incorporate additional complexity and biological realism in a stepwise fashion (Fig 1B). In all these models we follow the entire bacterial biomass rather than single cells. For our purposes here and in contrast to previous work [23], we can simplify the model by assuming that there is negligible heterogeneity between single cells. Table 2 lists all parameters and variables of these models.

**Table 2:** Explanation of variables, constants and parameters.

Variables		Parameters and constants	
*A*_e_	Extracellular antibiotic	*k*_f_	Rate of forward reaction (binding of drug to target)
*A*_i_	Intracellular antibiotic	*k*_r_	Rate of backward reaction (unbinding of drug from target)
*T*	Free target	*p*	Permeability coefficient bacterial membrane (per bacterium)
*AT*	Bound target	*f*_c_	Fraction of free target at MIC
*AU*	Unspecifically bound antibiotic	*n*_A_	Avogadro constant (6.02 x 10^23^)
		*V*_i_	Intracellular volume (~10^-15^L/ bacterial cell)
		*K*	Carrying capacity (10^9^ bacteria/ml = 10^12^/L)
		*k*_u,f_	Rate of unspecific forward reaction
		*k*_u,r_	Rate of unspecific backward reaction

## Model 1: Drug-Target Binding Only

To build our understanding of the drug-target reaction kinetics as antibiotic concentrations fluctuate within a host, Model 1 focuses only on the drug-target binding that occurs after exposure and withdrawal of an antibiotic. For Model 1 we make the following simplifying assumptions (which are subsequently relaxed in Models 2 and 3):

1. During the time of exposure, the antibiotic concentration remains constant (assumption relaxed in model 2)
2. There is no concentration gradient or diffusion barrier between the medium and the intracellular space (assumption relaxed in model 2)
3. Target molecules are not reproduced by the bacteria (assumption relaxed in model 3)
4. Target binding is described with a single apparent association rate (and, where relevant, dissociation rate); this assumption partially relaxed in model 3, where we also consider unspecific (i.e. off-target) binding.

The chemical reaction of antibiotics with their targets is described by the following equation: *A*+*T* ⇌ *AT*. The intracellular antibiotic molecules *A* react with target molecules *T* with a rate *k*_*f*_ and form an antibiotic-target molecule complex. If the reaction is reversible, the complex dissociates with a rate *k*_*r*_, leading to a dynamic equilibrium.

The dynamics of this system are governed by the concentrations *[A]*, *[T]*, *[AT]* rather than the absolute number of molecules. We assume that the total concentration of target/cell *[T*_*0*_*]* is constant. In this case, the concentration of free target can be described as [*T*] = [*T*_0_] − [*AT*]. Assuming that cells are treatment-naïve, i.e. there are no bound target molecules at the beginning, the kinetics of antibiotic-target reaction can then be described by a single differential equation, which can be simplified if we assume the intracellular antibiotic concentration [*A*] is constant:

$$\frac{d[AT]}{dt}=k_{f}[A](\left[T_{0}]-[AT\right])-k_{r}[AT]$$ (1)

and solved as:

$$[AT](t)=\frac{k_{f}[A][T_{0}](1-e^{-{(k}_{r}+k_{f}[A])t})}{k_{r}+k_{f}[A]}$$ (2)

At a certain point, the fraction of bound target reaches a critical threshold at which the net growth of the bacterial population is zero. In this framework, the MIC is characterized as the minimal antibiotic concentration at which this critical percentage of bound target, *f*_c_, is reached. Thus, the MIC is the antibiotic concentration at which the equilibrium fraction of bound antibiotic is exactly *f*_c_: i.e.

$\frac{{\left[AT\right]}_{MIC}}{\left[T_{0}\right]}=f_{c}$. After simplifying, this yields:

$$MIC=\frac{K_{D}f_{c}}{1-f_{c}}$$ (3)

with the affinity constant $K_{D}=\frac{k_{r}}{k_{f}}$.

Expressing all antibiotic concentrations as fold-MIC (*xMIC)* and thereby replacing [A] with $MIC\;\frac{K_{D}f_{c}}{1-f_{c}}$, Eq (2) can then be transformed:

$$[AT](t)=\frac{f_{c}T_{0}xMIC}{1-f_{c}(1-xMIC)}\left(1-e^{-\frac{k_{r}(1-f_{c}(1-xMIC))t}{1-f_{c}}}\right)$$ (4)

The time to the onset of antibiotic action, i.e. the delay until the fraction of bound target first exceeds *f*_c_ after antibiotic administration, can be expressed as:

$$t_{onset}=\frac{f_{c}-1}{k_{r}(1+f_{c}(xMIC-1))}log\;\left(-\frac{\left(xMIC-1)(f_{c}-1\right)}{xMIC}\right)$$ (5)

## Model 2: Drug-Target Binding with Diffusion Barrier and Bolus Injection

We next extend Model 1 to allow for fluctuating antibiotic concentrations after bolus dosing and to account for diffusion across the bacterial cell envelope. (These extensions effectively relax the first two assumptions for Model 1).

### Model 2a: Ampicillin

Model 2 includes the following compartments: *A*_e_, the number of extracellular antibiotic molecules, A_i_, the number of intracellular antibiotic molecules, *T*, the number of free target molecules, and *AT*, the number of drug-target complexes. For bolus injections, the model is described by the following set of equations:

$$\begin{array}{l} \frac{dA_{e}}{dt}=-\frac{\ln\left(2\right)}{t_{cl}}A_{e}-p\left(A_{e}\frac{V_{i}}{V_{e}}-A_{i}\right)\\ \frac{dA_{i}}{dt}=p\left(A_{e}\frac{V_{i}}{V_{e}}-A_{i}\right)-\frac{k_{f}}{n_{A}V_{i}}A_{i}T+k_{r}AT\\ \;\frac{dT_{}}{dt}=-\frac{k_{f}}{n_{A}V_{i}}A_{i}T+k_{r}AT\\ \frac{dAT}{dt}=\frac{k_{f}}{n_{A}V_{i}}A_{i}T-k_{r}AT \end{array}$$ (6)

To model an alternative drug administration approach in which the antibiotic concentration is maintained at a constant level *c* and after a specified time (*t*_end_) is assumed to fall instantaneously to 0 (i.e. intravenous dosing), the extracellular antibiotic concentration is given by:

$$A_{e}=\left\{ \begin{array}{c} c\;\text{for}\;t<t_{end}\\ 0\;\text{for}\;t\geq t_{end} \end{array}\right.$$ (7)

We express the antibiotic concentration as fold-MIC (*xMIC*) using Eq (3). The terms describing the chemical kinetics of drug-target reaction are equivalent to Eq (1). In addition, we describe the diffusion through the cell envelope with a permeability coefficient *p* depending on the concentration difference inside and outside of the bacterial cells and the clearance of the extracellular antibiotic; its half-life is *t*_cl_. In our simulations, drug binding and diffusion from extra- to intracellular space changes the dynamics of external drug concentrations negligibly, even though this may change at very high bacterial loads with a high number of targets per cell [69].

### Model 2B: Isoniazid

Here, we use the same equations and parameters as in Figure 7 in [23], extended by diffusion across the cell envelope and a decay term that describes the elimination of the drug from the blood after a bolus injection with t_1/2_. In the case of the prodrug isoniazid (INH), target binding occurs after drug activation to the adduct INH•NAD (equivalent to A before) which depends on NAD content and oxygen saturation. Here, we focus on INH binding to the enoyl reductase InhA, which is then present in its inactive form InhA_i_. Assuming NAD and target molecule concentration as well as oxygen saturation remain constant, the number of molecules in each compartment is described by the following set of equations:

$$\begin{array}{l} \frac{{dINH}_{e}}{dt}=-\frac{\ln\left(2\right)}{t_{cl}}{INH}_{e}-p\left({INH}_{e}\frac{V_{i}}{V_{e}}-{INH}_{i}\right)\\ \frac{{dINH}_{i}}{dt}=p\left({INH}_{e}\frac{V_{i}}{V_{e}}-{INH}_{i}\right)-k_{NAD,O_{2}}{INH}_{i}\\ \frac{dINH∙NAD}{dt}=k_{NAD,O_{2}}{INH}_{i}-\frac{k_{f}}{n_{A}V_{i}}INH∙NAD\;InhA+k_{r}\;{InhA}_{i}\\ \frac{d{InhA}_{i}}{dt}=\frac{k_{f}}{n_{A}V_{i}}INH∙NAD\;InhA-k_{r}{InhA}_{i} \end{array}$$ (8)

This set of equations is based on (6) and we additionally model prodrug activation.

## Model 3: Including Unspecific Binding and Bacterial Replication

Finally, Model 3 expands on Model 2 by allowing the reproduction of target molecules that would occur as a result of bacterial replication and also allows for unspecific binding. (This extension relaxes assumption 3 and partially relaxes assumption 4 in the list provided above.) This model describes antibiotics that only suppress bacterial growth but do not increase bacterial killing (i.e. bacteriostatic agents). For bacteriostatic translation inhibitors such as tetracycline, the bacterial replication rate depends linearly on the fraction of free ribosomes [70]. We therefore assume that the bacterial growth rate *r* is proportional to $f_{free}=\frac{\left[T\right]}{\left[T]+[AT\right]}$ above *f*_*f*_ = 1- *f*_*c*_ and that there is no growth when the fraction of free ribosomes falls below this critical threshold:

$$r(f_{free})=\left\{ \begin{array}{c} \;0\;\text{for}\;f_{free}<\;f_{f}\\ r_{no\;drug}\frac{1}{1-f_{f}}\;(f_{free}\text{-}f_{f})\;\text{for}\;f_{free}>\;f_{f} \end{array}\right.$$ (9)

Here, we track bacterial cells *B* (scaled in number of cells per liter) that can reproduce until they reach a maximal carrying capacity *K*, the extracellular and intracellular number of antibiotics *A*_*e*_ and *A*_*i*_, and the intracellular concentration of drug-target complexes *AT* and unspecifically bound antibiotic *AU*. The rates *k*_*f*_ and *k*_*r*_ describe specific binding and dissociation, the rates *k*_*u*,*f*_ and *k*_*u*,*r*_ describe the rates for unspecific binding and dissociation. Data indicate that the total number of ribosomes increases linearly with cell volume; this means that the intracellular concentration within a single cell between the time of its “birth” and the split into two daughter cells remains relatively constant [31]. We can therefore write the number of free target molecules as *T* = *BT*_0_ − *AT* with T_0_ describing the fixed number of total target molecules per cell. The growth of bacteria exposed to sub-MIC concentrations of a translation inhibitor can then be described by the following set of differential equations (note that we are again following molecules, not molar concentrations):

$$\begin{array}{l} \frac{dB}{dt}=r(B,{\;T}_{0},\;[AT],f_{c})\;B\;\left(1-\frac{B}{K}\right)\\ \frac{dA_{e}}{dt}=-\frac{\ln\left(2\right)}{t_{cl}}A_{e}-p\left(A_{e}\frac{V_{i}}{V_{e}}-A_{i}\right)\\ \frac{dA_{i}}{dt}=p\left(A_{e}\frac{V_{i}}{V_{e}}-A_{i}\right)-\frac{k_{f}}{n_{A}V_{i}}A_{i}T+k_{r}AT\;-k_{u,f}\;A_{i}\;B+\;k_{u,r}AU\\ \frac{dAT}{dt}=-\frac{k_{f}}{n_{A}V_{i}}A_{i}T+k_{r}AT\\ \frac{dAU}{dt}=k_{u,f}\;A_{i}\;B-\;k_{u,r}AU \end{array}$$ (10)

Again, this equation is based on (6), in addition, we model bacterial population biology by following the total amount of bacteria B.

## Supporting Information

S1 Fig Analysis of single cell time-lapse microscopy data for *E*. *coli* MG1655 cells exposed to ampicillin. Cells were grown in a flow chamber supplied with medium without antibiotics for 4 h (pre-phase, first section), exposed to antibiotic for 16h (peri-phase, middle section), followed by growth in medium that did not contain antibiotics for 4h (post-phase, last section)). **(A)** replication rate h^-1^; **(B)** elongation rate in pixel/min. Experimental mean in 5 min intervals (thick black line), experimental mean for entire pre-, peri- and post-exposure period (blue line), experimental minimum and maximum in 5 min intervals (thin, dotted black line), experimental minimum and maximum for entire pre-, peri- and post-exposure period (blue shaded area). 20 bacteria were observed and exposed to 6 mg/L ampicillin (0.8x MIC) for 16 h. For details see [23, 71].
(TIF)

S2 Fig Drug half-life and efficacy. The x-axes show the time after initiation of antibiotic therapy in hours, the y-axes the current antibiotic concentration in fold MIC (black, left side) and the % bound target (violet, right side). The green line shows the antibiotic concentration outside and inside the cell (assuming that there is a negligible diffusion barrier), the violet line shows the amount of bound target (refers to y-axis on the right). The grey area indicates that either the antibiotic concentration is below MIC or the fraction of bound target is below the inhibitory threshold *f*_c_. The dotted vertical lines indicate beginning and end of antibiotic action. The time the antibiotic is active, *T*_C>MIC_ and *AUC* are given in the figure title. Graphs in the first column depict bolus injections with an initial antibiotic concentration of 50MIC and a half-life of 1/2h, the half-life in the second column is 1h and the half-life in the third column is 2h. All graphs show drug-target binding expected based on physicochemical characteristics of ampicillin drug-target binding from the literature (Table 1, compare to Fig 5A). **(A)** Includes no diffusion barrier, **(B)** includes a diffusion barrier with *p* = 10^−4^, and **(C)** an diffusion barrier with *p* = 10^−5^.
(PDF)

S3 Fig Biochemical properties shape antibiotic pharmacodynamics. The x-axes show the time after initiation of antibiotic therapy in hours, the y-axes the current antibiotic concentration in fold MIC (black, left side) and the % bound target (violet, right side). The green line shows the antibiotic concentration outside and inside the cell (assuming that there is a negligible diffusion barrier), the violet line shows the amount of bound target (refers to y-axis on the right). The grey area indicates that either the antibiotic concentration is below MIC or the fraction of bound target is below the inhibitory threshold *f*_c_. The dotted vertical lines indicate beginning and end of antibiotic action. Graphs in the first column depict bolus injections with an initial antibiotic concentration of 50MIC and a half-life of 1h. The second column shows a hypothetical dosing regimen with a constant concentration just above the MIC (1.01 MIC) that has the same *T*_C>MIC_ as in the first column. The third column shows a hypothetical dosing regimen with a constant concentration just above the MIC (1.01 MIC) that has the equivalent area under the curve (AUC) as in the first column. Note the different timescale in the third column. All graphs show drug-target binding expected based on physicochemical characteristics of ampicillin drug-target binding from the literature (Table 1, compare to Fig 5A) with the following modifications. **(A)** *t*_bound_ = 19h 15 min (*k*_r_ = 10^−5^). **(B)** *f*_c_ = 10%.
(TIF)

S4 Fig This figure shows the output of a multivariate regression of *T*_C>MIC_, *AUC* and *C*_max_ with t_onset_. We used the Akaike Information Criterion as implemented in the function step() in R to identify the model that best describes the data. This function would drop all explanatory variables that do not improve model quality. While all explanatory variables (*T*_C>MIC_, *AUC* and *C*_max_)were kept, only *C*_max_ is significantly correlated with t_onset_. The statistical programming software R was used, output of the function summary(step(lm())).
(PNG)

S1 Movie Modeled effect of bolus injection of ampicillin on individual bacterial cells and bacterial populations. These graphs show the expected dynamics of antibiotic-target reaction according to model 2 with all parameters adapted to ampicillin (see Table 1). The left side shows a cartoon of drug-target binding and how this affects bacterial viability. The amount of different classes of molecules (bound, extracellular or intracellular antibiotic) is adapted such that there are 10 target molecules/cell and at the peak 10 extracellular antibiotic molecules. Upon reaching the required threshold, cells turn grey (dead or non-replicating). Surviving cells or cells that were not replicating, but also not killed may restore full viability (lose the grey shade) after the amount of bound antibiotics falls below the required threshold again. On the right side, the Fig 5A, left panel is shown and the timecourse corresponding to the cartoon is highlighted. The x-axis shows the time after initiation of antibiotic therapy in hours, the y-axes the current antibiotic concentration in fold MIC (black, left side) and the % bound target (violet, right side). The green line shows the antibiotic concentration outside and the red inside the cell (both refer to the y-axis on the left), the violet line shows the amount of bound target (refers to y-axis on the right). The grey area indicates that either the antibiotic concentration is below MIC or the fraction of bound target is below the inhibitory threshold *f*_c_. The dotted vertical lines indicate beginning and end of antibiotic action.
(MOV)

S2 Movie Modeled effect of a constant concentration of ampicillin on individual bacterial cells and bacterial populations. The setup is the same is in movie 1, but this movie corresponds to Fig 5A, middle panel. The amount of molecules is adapted to movie 1, with exception of the amount of extracellular antibiotic. Compared to movie 1, this concentration would amount to less than 1 molecule, and for clarity, we chose to depict one molecule.
(MOV)

S3 Movie Modeled effect of bolus injection of an antibiotic with lower threshold and intracellular target on individual bacterial cells and bacterial populations. The setup is the same as in movie 1, but we assume a diffusion barrier of *p* = 10^−4^ /s and a threshold *f*_*c*_ of 10%.
(MOV)

S4 Movie Modeled effect of a constant concentration of an antibiotic with lower threshold and intracellular target on individual bacterial cells and bacterial populations. The setup is the same as in movie 1, but we assume a diffusion barrier of *p* = 10^−4^ /s and a threshold *f*_*c*_ of 10%.
(MOV)

S1 Table This table shows all parameter combinations used for Fig 6. Note that for combinations of low *C*_max_ and short half-lives, the critical threshold could not be reached, i.e. the dosing strategy did not have an antibacterial effect. Values are given in hours.
(DOC)
