# Supplementary figures and images for: Using Chemical Reaction Kinetics to Predict Optimal Antibiotic Treatment Strategies
Source: PLoS Comput Biol. 2017 Jan 6;13(1):e1005321. doi: 10.1371/journal.pcbi.1005321 (PMC5257006; doi:10.1371/journal.pcbi.1005321)

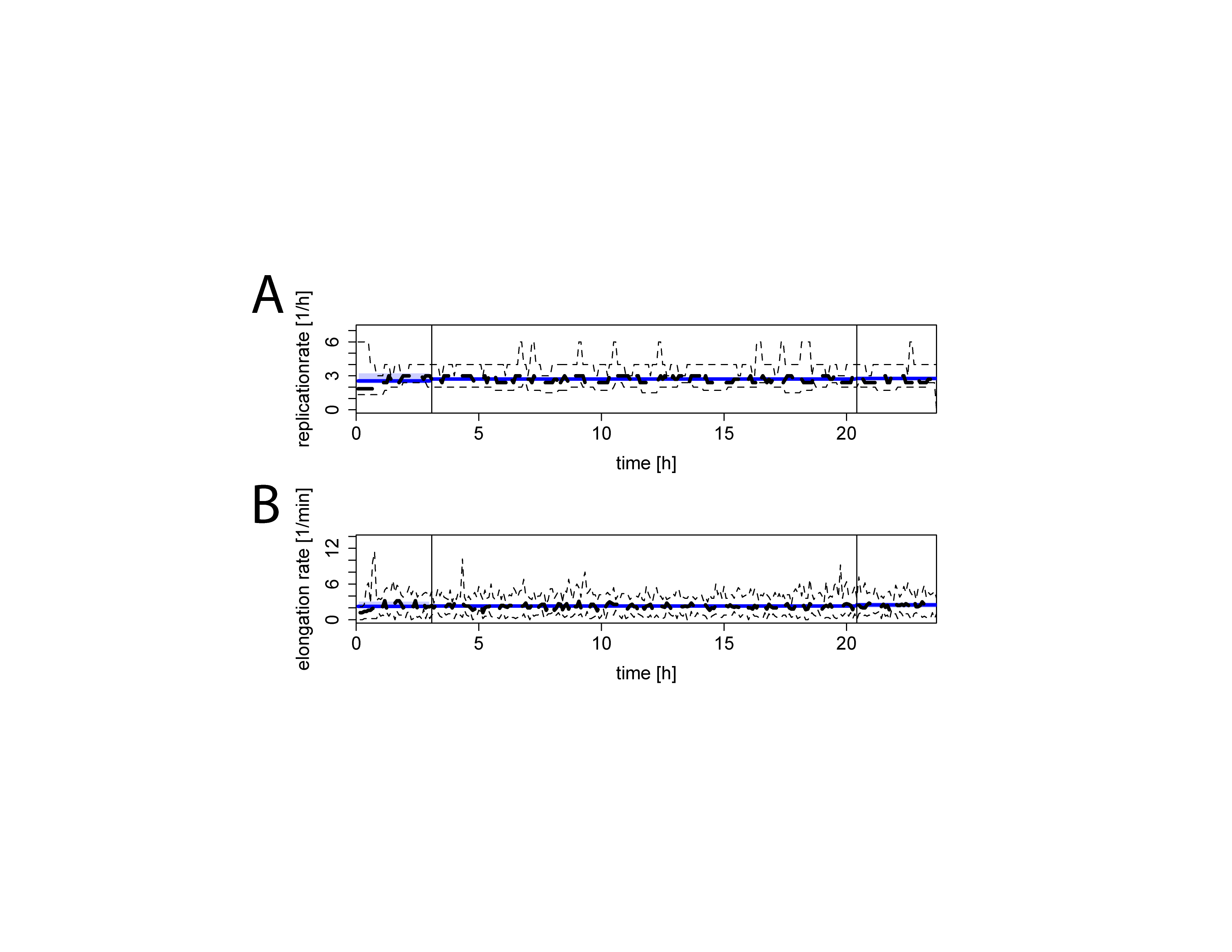

Supplement: S1 Fig — Cells were grown in a flow chamber supplied with medium without antibiotics for 4 h (pre-phase, first section), exposed to antibiotic for 16h (peri-phase, middle section), followed by growth in medium that did not contain antibiotics for 4h (post-phase, last section)). (A) replication rate h-1; (B) elongation rate in pixel/min. Experimental mean in 5 min intervals (thick black line), experimental mean for entire pre-, peri- and post-exposure period (blue line), experimental minimum and maximum in 5 min intervals (thin, dotted black line), experimental minimum and maximum for entire pre-, peri- and post-exposure period (blue shaded area). 20 bacteria were observed and exposed to 6 mg/L ampicillin (0.8x MIC) for 16 h. For details see [23, 71]. (TIF) [file pcbi.1005321.s001.tif]

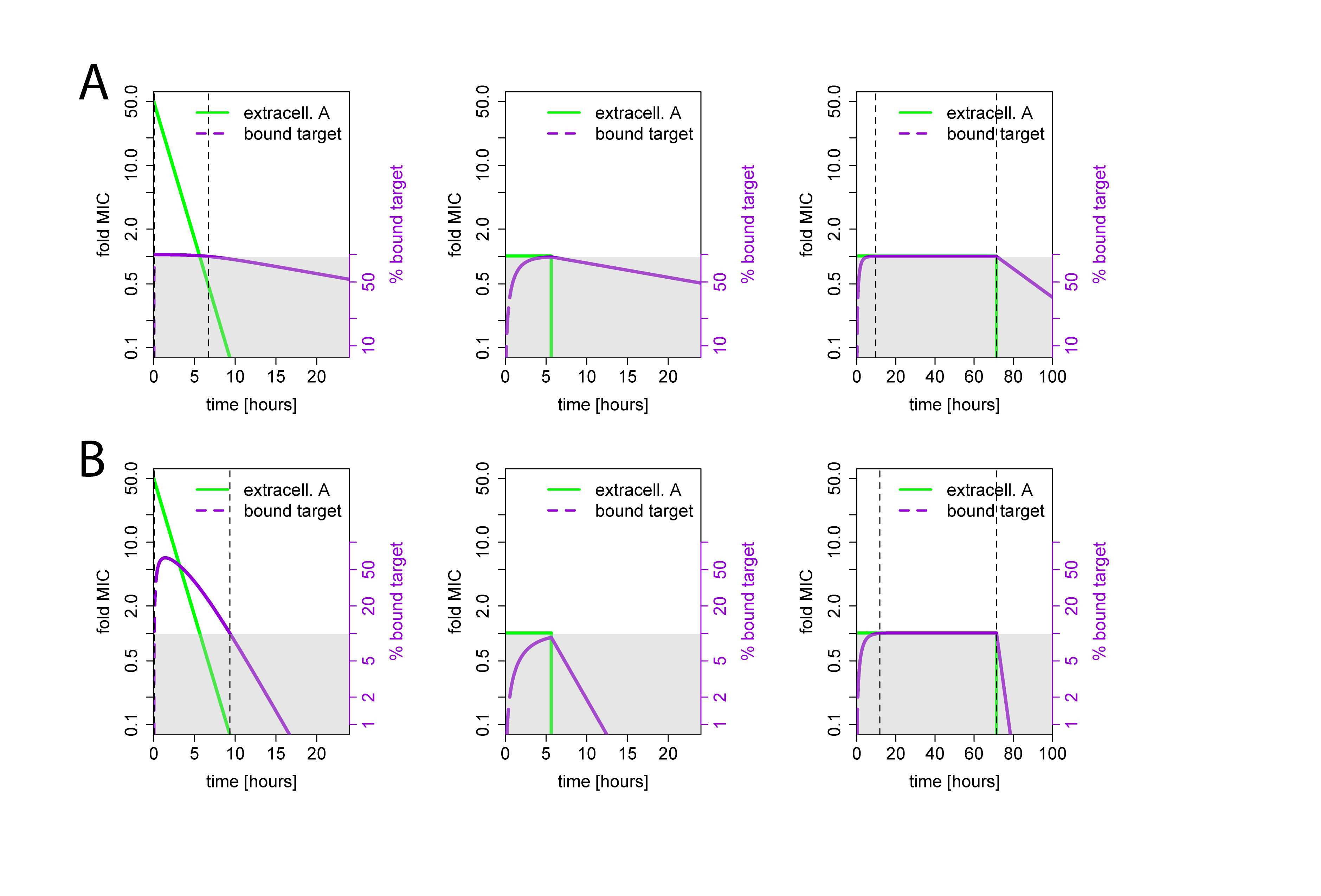

Supplement: S3 Fig — The x-axes show the time after initiation of antibiotic therapy in hours, the y-axes the current antibiotic concentration in fold MIC (black, left side) and the % bound target (violet, right side). The green line shows the antibiotic concentration outside and inside the cell (assuming that there is a negligible diffusion barrier), the violet line shows the amount of bound target (refers to y-axis on the right). The grey area indicates that either the antibiotic concentration is below MIC or the fraction of bound target is below the inhibitory threshold fc. The dotted vertical lines indicate beginning and end of antibiotic action. Graphs in the first column depict bolus injections with an initial antibiotic concentration of 50MIC and a half-life of 1h. The second column shows a hypothetical dosing regimen with a constant concentration just above the MIC (1.01 MIC) that has the same TC>MIC as in the first column. The third column shows a hypothetical dosing regimen with a constant concentration just above the MIC (1.01 MIC) that has the equivalent area under the curve (AUC) as in the first column. Note the different timescale in the third column. All graphs show drug-target binding expected based on physicochemical characteristics of ampicillin drug-target binding from the literature (Table 1, compare to Fig 5A) with the following modifications. (A) tbound = 19h 15 min (kr = 10−5). (B) fc = 10%. (TIF) [file pcbi.1005321.s003.tif]

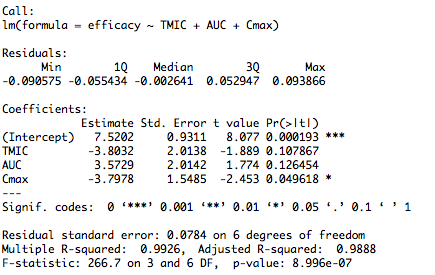

Supplement: S4 Fig — We used the Akaike Information Criterion as implemented in the function step() in R to identify the model that best describes the data. This function would drop all explanatory variables that do not improve model quality. While all explanatory variables (TC>MIC, AUC and Cmax)were kept, only Cmax is significantly correlated with tonset. The statistical programming software R was used, output of the function summary(step(lm())). (PNG) [file pcbi.1005321.s004.png]
