# Supplementary material for: Using Chemical Reaction Kinetics to Predict Optimal Antibiotic Treatment Strategies
Source: PLoS Comput Biol. 2017 Jan 6;13(1):e1005321. doi: 10.1371/journal.pcbi.1005321 (PMC5257006; doi:10.1371/journal.pcbi.1005321)
Supplement: S2 Fig — The x-axes show the time after initiation of antibiotic therapy in hours, the y-axes the current antibiotic concentration in fold MIC (black, left side) and the % bound target (violet, right side). The green line shows the antibiotic concentration outside and inside the cell (assuming that there is a negligible diffusion barrier), the violet line shows the amount of bound target (refers to y-axis on the right). The grey area indicates that either the antibiotic concentration is below MIC or the fraction of bound target is below the inhibitory threshold fc. The dotted vertical lines indicate beginning and end of antibiotic action. The time the antibiotic is active, TC>MIC and AUC are given in the figure title. Graphs in the first column depict bolus injections with an initial antibiotic concentration of 50MIC and a half-life of 1/2h, the half-life in the second column is 1h and the half-life in the third column is 2h. All graphs show drug-target binding expected based on physicochemical characteristics of ampicillin drug-target binding from the literature (Table 1, compare to Fig 5A). (A) Includes no diffusion barrier, (B) includes a diffusion barrier with p = 10−4, and (C) an diffusion barrier with p = 10−5. (PDF) [file pcbi.1005321.s002.pdf]

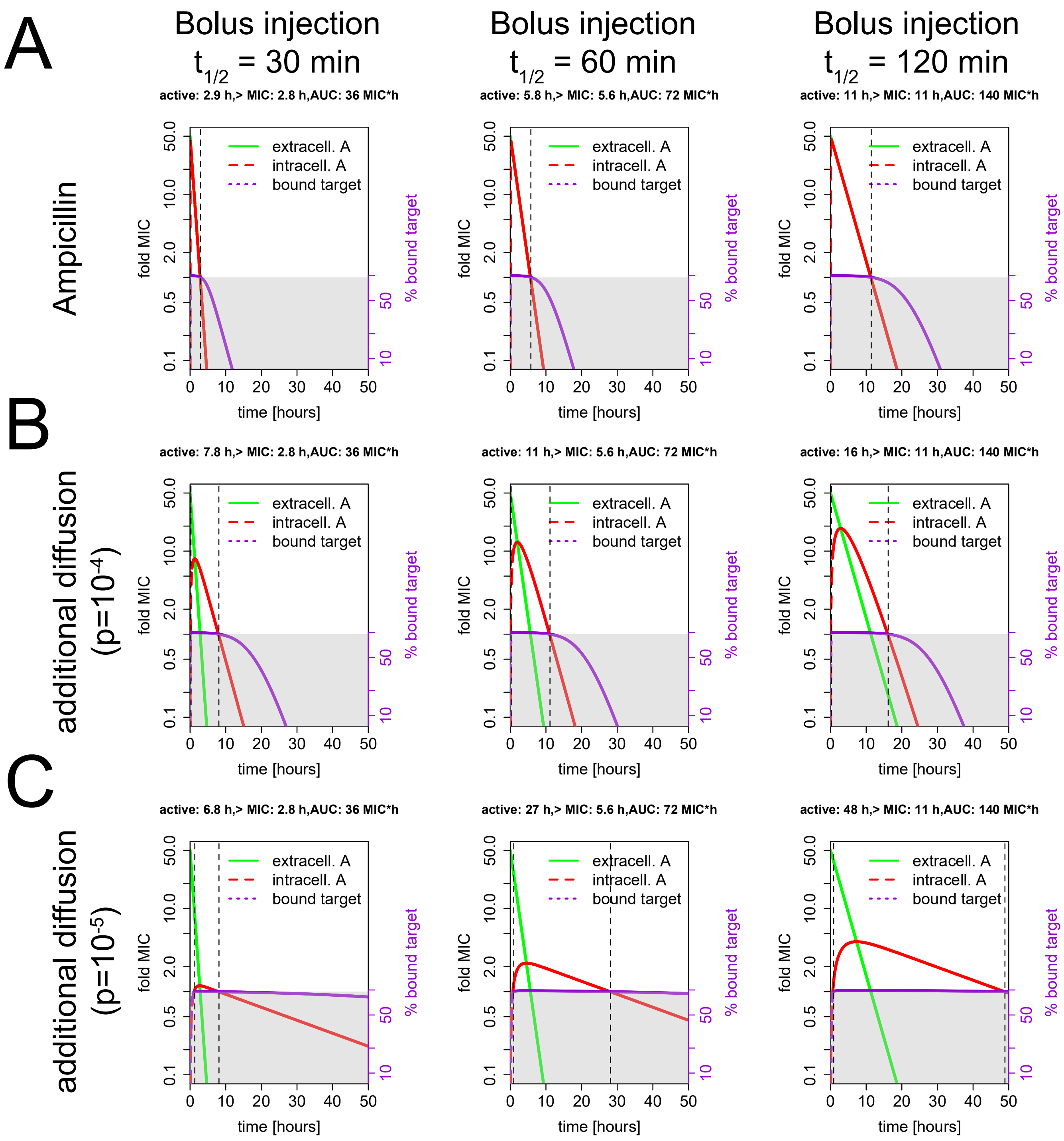
A
B
C
Bolus injection
$t_{1/2}$ = 30 min
Bolus injection
$t_{1/2}$ = 60 min
Bolus injection
$t_{1/2}$ = 120 min
Ampicillin
additional diffusion
($p=10^{-4}$)
additional diffusion
($p=10^{-5}$)
active: 2.9 h,> MIC: 2.8 h,AUC: 36 MIC*h
active: 5.8 h,> MIC: 5.6 h,AUC: 72 MIC*h
active: 11 h,> MIC: 11 h,AUC: 140 MIC*h
active: 7.8 h,> MIC: 2.8 h,AUC: 36 MIC*h
active: 11 h,> MIC: 5.6 h,AUC: 72 MIC*h
active: 16 h,> MIC: 11 h,AUC: 140 MIC*h
active: 6.8 h,> MIC: 2.8 h,AUC: 36 MIC*h
active: 27 h,> MIC: 5.6 h,AUC: 72 MIC*h
active: 48 h,> MIC: 11 h,AUC: 140 MIC*h
extracell. A
intracell. A
bound target
fold MIC
% bound target
time [hours]
0.1
0.5
2.0
10.0
50.0
10
50
0
10
20
30
40
50
