# Supplementary material for: Using Chemical Reaction Kinetics to Predict Optimal Antibiotic Treatment Strategies
Source: PLoS Comput Biol. 2017 Jan 6;13(1):e1005321. doi: 10.1371/journal.pcbi.1005321 (PMC5257006; doi:10.1371/journal.pcbi.1005321)
Supplement: S1 Table — Note that for combinations of low Cmax and short half-lives, the critical threshold could not be reached, i.e. the dosing strategy did not have an antibacterial effect. Values are given in hours. (DOC) [file pcbi.1005321.s009.doc]

|  | **t_1/2_ = 0.5 h** | **t_1/2_ = 1 h** | **t_1/2_ = 2 h** | **t_1/2_ = 4 h** |
| --- | --- | --- | --- | --- |
| 25 MIC |  |  |  | 7.0 |
| 50 MIC |  |  | 3.6 | 2.7 |
| 100 MIC |  | 1.9 | 1.4 | 1.3 |
| 200 MIC | 1.1 | 0.8 | 0.8 | 0.7 |
